# Myeloid differentiation-associated autophagy drives venetoclax resistance in AML

**DOI:** 10.21203/rs.3.rs-10617934/v1

**Published:** 2026-08-10

**Authors:** Johannes Menzel, Mark A. Gregory, Daniela Ortiz Chavez, Jacqueline S. Thorburn, Marco De Dominici, Daniel A. Pollyea, Craig T. Jordan, Andrew Thorburn, James DeGregori

**Affiliations:** 1 The Department of Biochemistry and Molecular Genetics, University of Colorado Anschutz Medical Campus, Aurora, CO, USA.; 2 The Department of Pharmacology, University of Colorado Anschutz Medical Campus, Aurora, CO, USA.; 3 The Department of Medicine, Division of Hematology, University of Colorado Anschutz Medical Campus, Aurora, CO, USA.; 4 The Department of Immunology and Microbiology, University of Colorado Anschutz Medical Campus, Aurora, CO, USA.

## Abstract

Acute myeloid leukemia (AML) is a complex disease that often displays phenotypic and genotypic heterogeneity, where subclones are either refractory or frequently become resistant to treatment. Several studies have shown that the developmental stage of leukemia correlates with the response to therapies combining the BCL2 inhibitor venetoclax (Ven) with hypomethylating agents, such as azacitidine (Aza), where phenotypically primitive AML cells are sensitive and monocytic cells are more resistant. Here we show that resistant monocytic subclones have higher autophagic flux compared to sensitive primitive subclones in AMLs of mixed phenotype. Induction of AML myeloid differentiation with inecalcitol, a potent vitamin D3 analogue and vitamin D receptor (VDR) agonist, increases autophagic flux and Ven/Aza resistance. Pharmacological inhibition or genetic perturbation of autophagy negates differentiation-induced Ven/Aza resistance. We show that this myeloid differentiation-associated autophagy increases mitochondrial reserve capacity and metabolic flexibility. This work shows that autophagy-dependent metabolic rewiring during AML differentiation contributes to Ven/Aza therapy resistance.

## Introduction

Acute myeloid leukemia (AML) is a cancer of the bone marrow and blood, characterized by abnormal cells of the myeloid lineage that grow out and interfere with normal blood cell production. Selective inhibition of BCL2 using the drug venetoclax has been shown to suppress oxidative phosphorylation (OXPHOS) and impair survival of leukemia stem cells (LSC) in AML (1). Venetoclax (Ven) used in combination with a hypomethylating agent (HMA), such as azacitidine (Aza) or decitabine, now represents the standard of care regimen for newly diagnosed older or unfit adults with AML. Although Ven/HMA therapy has greatly improved survival compared with HMA monotherapy, most patients will eventually relapse, and outcomes for relapsed/refractory disease remain poor. The developmental stage of leukemia often correlates with the response to Ven/Aza therapy, with phenotypically primitive AML cells being sensitive and cells with monocytic differentiation being more resistant (1–5). How this developmental stage or differentiation state in AML interplays with autophagy, mitochondrial metabolism, and response to Ven/Aza is not well understood.

One hallmark of cancer is the reprogramming of metabolism to support survival and proliferation (6). Normal hematopoietic stem cells (HSC) avoid reactive oxygen species (ROS)-producing OXPHOS and instead use anaerobic glycolysis for their routine energy needs (7, 8). In contrast, LSCs rely on mitochondrial OXPHOS for survival, while maintaining ROS levels at low non-toxic levels (9). AMLs exhibit a variety of changes in amino acid, pyrimidine, and fatty acid metabolism to support OXPHOS (10). Despite having higher respiratory activity, AML mitochondria have lower coupling efficiency and spare reserve capacity making them susceptible to oxidative metabolic stress (11).

Autophagy is a process that degrades and recycles molecular components and cellular organelles, used by cells as a source of molecular components and energy during starvation, and can also be invoked to selectively remodel the proteome during cellular differentiation (12–16). Many studies have shown that autophagy induction is linked to a pro-survival response during chemotherapy to suppress treatment efficiency in AML (17–21). Autophagy has been implicated as an important source of metabolic substrates that fuel a variety of cancers including AML (22–26). Fatty acid oxidation in particular has been shown to be a crucial catabolic pathway for AML (27, 28). Autophagy has been shown to directly supply free fatty acids to mitochondria through the degradation of lipid droplets in AML cells, but not in normal hematopoietic cells (29). The importance of mitochondrial metabolism for maintaining cell survival is becoming clearly evident, suggesting new therapeutic avenues for AML (30–33). Here, we show that AML differentiation is associated with an increase in autophagy, improved mitochondrial functional reserve and metabolic flexibility, and venetoclax resistance, all of which are dependent on autophagy.

## Results

### Monocytic AML subclones are associated with higher autophagic flux, Ven/Aza resistance, and elevated inflammatory signaling.

AML is a heterogeneous disease. In addition to inter-patient heterogeneity, individual patients often present complex clonal heterogeneity where subpopulations exhibit features along a spectrum of myeloid differentiation. More differentiated, monocytic AMLs and subpopulations tend to be more resistant to Ven/Aza. To demonstrate this common feature of Ven resistance in monocytic subpopulations, a primary AML with mixed primitive and monocytic subpopulations, AML1, was treated with either vehicle or 500 nM Ven and 1.5 μM Aza for 72 hours, then stained with antibodies against myeloid surface markers to distinguish monocytic lineage AML cells (CD11b^+^, CD14^+^) and more primitive AML cells (CD11b^neg^, CD14^neg^), together with DAPI to measure cell viability. Analysis by flow cytometry revealed substantially greater cell loss within the more primitive subpopulation (Figure 1A). See Supplemental Table 1 for characteristics of patient samples used in this study.

Autophagy has been shown to be involved in drug resistance and can modulate cell death sensitivities (34, 35). Additionally, autophagy not only provides a source of energy and salvaged molecular components but also has a role in proteomic remodeling during cellular differentiation. In particular, autophagy is critical for the proper development of functional macrophages from monocytes (16, 36, 37). Notably, gene expression data from Ven/Aza resistant monocytic versus Ven/Aza sensitive primitive AML LSCs show elevated transcript levels from several autophagy related genes, including ATG3, ATG7, and TFEB (Figure 1B) (3).

We were interested in investigating the relationship between autophagy, AML differentiation, and death sensitivity to therapeutic agents. To determine whether monocytic subclones have higher autophagic flux, we utilized an autophagy reporter construct consisting of GFP linked to an LC3 motif that is actively targeted to the autophagosome and degraded in an autophagy-dependent manner, along with an internal control RFP lacking the LC3 motif (38). Using flow cytometry, the ratio of the mean fluorescent intensity (MFI) of RFP to GFP in cells is a measure of autophagic flux, where high RFP/GFP corresponds to high autophagy and low RFP/GFP to low autophagy. We introduced this autophagy reporter into AML cells via lentiviral transduction to assess the autophagic flux of various cell lines and primary AMLs. We then measured myeloid surface marker antigens using flow cytometry to gauge differentiation state and identify subpopulations within mixed primary AML. The more differentiated monocytic primary AML subpopulations with high CD11b and CD14 expression had higher autophagic flux across all samples tested (Figure 1C).

### Transient heterogeneity in autophagy influences Ven/Aza resistance and inflammatory signaling resembling features of more stable heterogeneity in primary AML subpopulations.

To further investigate the contribution of autophagy status to therapeutic sensitivity without potential confounding influences of AML heterogeneity (such as in differentiation status), we introduced our reporter into MOLM-13 AML cells and isolated single cell clones. Clones were grown out and then sorted into autophagy high and low populations for approximately the top and bottom 15% of RFP/GFP. We found the autophagy status of these sorted cells was transient and would converge to restore the original population distribution within 48 to 72 hours (Figure 1D). Importantly, despite this transient nature, these sorted populations exhibit differential sensitivity to drugs, with the ATG^HI^ cells (24 hours after sorting) exhibiting relative resistance to the apoptosis-inducing combination of Ven/Aza but sensitivity to the necroptosis-inducing combination of the caspase-8 inhibitor emricasan plus SMAC mimetic AZD5582 (Emr/Azd) (Figure 1E) (39). Thus, transient variation of autophagy within an AML population may contribute to opposing sensitivities to drugs that induce apoptosis and necroptosis.

RNA-seq analyses revealed striking gene expression differences between freshly sorted ATG^HI^ and ATG^Low^ MOLM-13 populations. The mTOR pathway, a negative regulator of autophagy, was decreased in ATG^Hi^ cells (p-adjust 5.6×10^−8^; NES −1.60) (Figure S1) (33), while multiple pathways related to inflammation and myeloid differentiation were increased (e.g. TNFA-Signaling-Via-NFkB; p-adjust 2.97×10^−7^; NES 1.87) (Figure 1F). These differences mirror those that distinguish Ven/Aza resistant monocytic AML from Ven/Aza sensitive primitive AML (Figure 1G and S1). Notably, both ATG^HI^ MOLM-13 cells and more monocytic patient AML cells exhibit reductions in Myc pathways – Myc has been demonstrated to suppress both differentiation (including myeloid) and autophagy, and to contribute to apoptotic sensitivity (40–42). Remarkably, differential drug sensitivities and associated gene expression patterns correlate well with respect to both these transient features seen in cell lines and stable features of primary AML subpopulations.

Cell cycle arrest, particularly in G0/G1, can be associated with increased drug resistance (43, 44). We asked whether transient changes in autophagy are associated with particular stages of the cell cycle. To assess cell cycle differences with respect to high or low autophagy, we stained cells harboring the autophagy reporter with Hoechst dye 33342 to measure DNA content and the cell cycle profile. Consistently across all cells tested, autophagy high cells were predominantly in G1/G0 of the cell cycle while autophagy low cells presented in G1, S, and G2 (Figure S1C and S1D). These data suggest that the cell cycle may contribute to transient changes in autophagy status. Still, autophagy low cells are present in all phases of the cell cycle. Moreover, stable autophagy high monocytic subpopulations in primary AML continue to cycle, indicating that the cell cycle state does not solely underlie autophagy status in AML cells (Figure S1E and S1F).

### Starvation-induced autophagy promotes Ven/Aza resistance.

We next sought to temporarily increase autophagic flux and evaluate changes in Ven/Aza sensitivity in primary AML. Starvation or nutrient deprivation is known to induce autophagy in cells. We resuspended primary AML cells harboring our autophagy reporter in Hank's Balanced Salt Solution (HBSS) for 24 hours to mimic nutrient deprivation and induce autophagy (Figure 2A, 2C, and 2E). We found that supplementing HBSS with FBS and glutamine improved survival and recovery of AML cells while still temporarily inducing autophagy (Figure S2F and S2G). We then resuspended these cells in Primary AML Media (PAM) with vehicle or Ven/Aza for an additional 48 hours to evaluate Ven/Aza sensitivity (Figure 2B, 2D, and 2F). This nutrient deprivation pretreatment increased autophagic flux and subsequent Ven/Aza resistance in all cells tested (Figure 2, and S2A – S2D). To substantiate that autophagy is responsible for increased Ven/Aza resistance we performed shRNA-mediated knock down of ATG7, a core autophagy gene, in MOLM-14 cells and evaluated drug sensitivity (Figure 2I and 2J). MOLM-14 cells with reduced ATG7 were significantly more sensitive to Ven and Ven/Aza. Since autophagy is critical for the proper development of functional macrophages from monocytes, we also asked if induction of autophagy via nutrient deprivation would influence or perhaps induces myeloid differentiation. When cells were pretreated with nutrient deprivation, we observed changes in myeloid surface marker expression; however, the changes were not consistent across different AMLs, as some primary AMLs exhibited increases in myeloid markers while other AMLs exhibited reductions in these markers (Figure 2G, 2H, and S2E). These data show that autophagy promotes resistance from Ven-induced cell death.

### Vitamin D receptor agonist inecalcitol promotes AML differentiation, autophagic flux and Venetoclax resistance.

To investigate whether autophagic flux and AML differentiation are functionally linked, we sought to induce differentiation pharmacologically. The active vitamin D metabolite 1α, 25-dihydroxyvitamin D_3_ (1,25 D3) is known to induce differentiation in hematopoietic cells, as is inecalcitol, a potent vitamin D3 analogue and vitamin D receptor (VDR) agonist (45–47). We discovered that inecalcitol strongly induced autophagy in most AML cells within 7 hours (Figure 3A). Given our results showing that monocytic AML and more differentiated subpopulations are frequently more resistant to Ven/Aza and have higher autophagic flux, we asked if inecalcitol-induced differentiation of AML cells would increase Ven/Aza resistance. We pre-treated AML cells with inecalcitol for 72 hours to allow for full differentiation and autophagy induction, then treated the cells with Ven/Aza for an additional 48 hours before assessing cell viability, autophagy, and myeloid surface markers via flow cytometry. AMLs showed variable increases of myeloid differentiation markers, autophagic flux, and Ven/Aza resistance in response to inecalcitol treatment (Figure 3B – 3G, and Figure S3). The extent of inecalcitol-induced differentiation correlates with the change in autophagy with an R^2^ value of 0.3988 for CD11b and 0.8269 for CD14 (Figure 3H and 3I), and the inecalcitol-induced change in autophagy correlates with relative survival in response to Ven/Aza with an R^2^ value of 0.6623, each p < 0.0001 (Figure 3J), revealing that the relative ability of inecalcitol to induce differentiation influences both autophagic flux and resistance to a pro-apoptotic therapy.

### Effects of inecalcitol on autophagic flux, cell cycle, and AML differentiation are reversible and depend on VDR.

Most AML cells tested showed a significant increase in autophagic flux within 7 hours of treatment with inecalcitol and continued to substantially increase flux and peak after 3 to 4 days of treatment (Figure 4A). This induction of autophagy was accompanied by an increase in myeloid surface makers, as expected (Figure 4B and 4C). We also observed a slowing of growth in many AMLs treated with inecalcitol (Figure 4D–4F), which is expected if cells are differentiating. Since cell cycle arrest and terminal differentiation are known to increase drug resistance, we tested whether inecalcitol treatment is causing permanent cell cycle arrest or terminally differentiated AMLs, thereby explaining increased Ven/Aza resistance. To determine the reversibility of inecalcitol-induced autophagy and differentiation, we treated several AMLs with 20nM inecalcitol for 3 days and then transitioned the cells into fresh media and allowed cells to recover for several days. We periodically assessed myeloid surface markers, autophagy, and cell cycle via flow cytometry. Three days of treatment with inecalcitol did induce autophagic flux (Figure 4A), increased myeloid surface marker expression (Figure 4B and 4C), and variably slowed the cell cycle (Figure 4D, 4E, and 4F, and S4), but all of these metrics eventually return to initial levels when cells were resuspended in fresh media without inecalcitol. These data indicate that inecalcitol can temporarily induce differentiation and autophagy in AML cells without causing permanent cell cycle arrest and terminal differentiation.

The VDR is the primary receptor for vitamin D. The response to inecalcitol is dependent on VDR, as CRISPR-mediated knockout of *VDR* abolished autophagy induction (Figure 4G) and myeloid marker expression (Figure 4H and 4I). Note that in Figure 4G, autophagy is measured only with the LC3-GFP, as we used a dTomato-labeled CRISPR construct to allow flow sorting of disrupted cells, and thus less GFP signal indicates greater autophagy. The protein disulfide isomerase family A member 3 (PDIA3), also known as 1, 25 D3-MARRS (1, 25 D3-membrane-associated, rapid response steroid-binding protein), acts as an alternative membrane-associated receptor (48). We tested the involvement of PDIA3 with respect to differentiation and autophagy induction and found that only ablation of *VDR*, not *PDIA3*, prevented a response to inecalcitol (Figure 4G, 4H, 4I, and S4). We used lentiviral delivered gRNA to effect CRISPR-mediated knockout of the *VDR* and *PDIA3* genes in MOLM-13 and MOLM-14 cells harboring the GFP-LC3 autophagy reporter and a Cas9-Blast constructs. We verified efficient disruption of *VDR* and *PDIA3* genes within the population of cells via TIDE sequence analysis across the CRISPR cut site (Figure S4H, S4I).

### The key regulator of hematopoietic differentiation CEBPA controls autophagy and drug resistance.

The transcription factor CCAAT Enhancer Binding Protein Alpha (encoded by *CEBPA*) is involved in hematopoietic differentiation and is often mutated in AML, where function-disrupting mutations induce a myeloid differentiation block (49–51). We asked whether manipulating *CEBPA* is sufficient to alter autophagy status, either by ectopic expression that can drive differentiation or CRISPR-mediated knockout. We used lentiviral delivered gRNA to achieve CRISPR-mediated knockout of the *CEBPA* gene in MOLM-14 cells harboring our autophagy reporter and a Cas9-expressing construct. We verified efficient disruption of the *CEBPA* gene within the population of cells via TIDE sequence analysis across the CRISPR cut site within *CEBPA*. We observed a reduction in autophagic flux and Ven resistance in *CEBPA*-mutated MOLM-14 cells (Figure 5A and 5B). Similarly, primary AML1 were first transduced with lentivirus containing a CRISPR construct against *CEBPA* followed by electroporation of Cas9 mRNA. *CEBPA* knock out was confirmed with Western blot and RT-qPCR measuring *CEBPA* expression (Figure S5A and S5B). These cells showed a reduction in both myeloid surface markers and Ven resistance (Figure 5C and 5D). We also observed an increase in resistance to the necroptosis-inducing drug combination of Emricasan/AZD5582 (Figure 5E). These data are consistent with our previous observations that transient autophagy low cells are more sensitive to therapies inducing apoptosis but more resistant to those inducing necroptosis. We also assessed the impact of ectopic expression of *CEBPA* in AML1 by transduction with a construct co-expressing *CEBPA* and GFP and observed increased myeloid differentiation markers and Ven/Aza resistance (Figure 5F and 5G). Thus, CEBPA-mediated increases in autophagy and differentiation promote Ven/Aza resistance and are consistent with our other findings where increased autophagy reduces sensitivity to apoptosis. We also evaluated how the knockout of *CEBPA* may influence the effects of inecalcitol, since the VDR and CEBPA have many overlapping or proximal binding sites within the genome (52–55). We induced differentiation and autophagy in our MOLM-14 CEBPA knock out cells by pre-treating with inecalcitol, followed by treatment of these cells with Ven or Ven/Aza for an additional 48 hours and assessment of viability via flow cytometry. CRISPR-mediated disruption of CEBPA did reduce, but did not completely prevent, the inecalcitol-induced protection to Ven/Aza (Figure 5H). Increased necroptosis resistance was again observed in the *CEBPA*-disrupted MOLM-14 cells, in this case in the context of inecalcitol treatment (Figure 5H). Taken together, *CEBPA* appears to contribute to myeloid differentiationassociated autophagy and be in part responsible for inecalcitol-induced Ven/Aza resistance.

### Blocking autophagy negates inecalcitol-induced drug protection in primary AML

To further test if autophagic flux is the process driving drug resistance, we used the autophagy inhibitor Bafilomycin A1 (BafA1) in combination with inecalcitol and drug treatments. Primary AML cells carrying the autophagy reporter were pretreated with inecalcitol for 72hrs then treated with Ven/Aza for an additional 48hrs with or without BafA1. Inecalcitol pre-treatment strongly induced autophagy (Figure 6A, 6C, and 6E), and increased Ven/Aza resistance (Figure 6B, 6D, and 6F); however, this protection was negated when autophagy was blocked with the autophagy inhibitor BafA1. These data support that myeloid differentiation-associated increases in Ven/Aza resistance are autophagy-dependent and that it is the increase in autophagic flux that provides protection against apoptosis induced by Ven/Aza.

### Differentiation-associated autophagy improves mitochondrial reserve capacity and metabolic flexibility.

Mitochondrial respiration is a crucial component of cellular metabolism that is often dysregulated in cancer. OXPHOS is a critical mitochondrial process that generates intracellular energy by metabolizing substrates such as glucose, glutamine, and fatty acids to generate electrons that flow through the respiratory chain complexes embedded within the mitochondrial membrane to create a transmembrane electrical potential (56–58). The energy stored in this gradient is used to drive adenosine triphosphate (ATP) production. AML cells have increased mitochondrial mass, low respiratory chain complex activities, and low spare reserve capacity compared with normal cells, leaving them particularly vulnerable to inhibitors of the respiratory chain complexes and oxidative stressors (9, 11, 32, 59, 60).

As OXPHOS and mitochondrial dynamics appear to play a significant role in maintenance and treatment resistance in myeloid leukemia, we asked how mitochondrial function may change during inecalcitol-induced differentiation and increased autophagy. We first assayed mitochondrial transmembrane potential and mass with TMRE (tetramethylrhodamine, ethyl ester) and MitoTracker Green stains, respectively, in AML cells treated with inecalcitol. TMRE is a cationic cell membrane permeable fluorescent dye that accumulates in intact mitochondria. Depolarized or inactive mitochondria exhibit decreased membrane potential, resulting in reduced TMRE accumulation. MitoTracker Green accumulates in mitochondria regardless of the mitochondrial membrane potential, making it a useful tool for measuring mitochondrial volume or mass and can be used to normalize the TMRE signal to total mitochondrial mass. Most AML cell lines and primary cells significantly increase mitochondrial transmembrane potential relative to mass in response to inecalcitol (Figure 7A, S7A, and S7B). Interestingly, mitochondrial mass also increased with inecalcitol (Figure S7B). These data suggest that inecalcitol promotes mitochondrial function alongside increased differentiation and autophagy.

To further investigate mitochondrial function, we used a Seahorse XF Mito Stress Test to measure the oxygen consumption rate (OCR) (Figure 7B) and extracellular acidification rate (ECAR) (Figure 7C) of AML1 and MV4–11 cells (Figure S7C and S7D) cells following inecalcitol-induced differentiation with or without autophagy inhibition with BafA1. In AML1, inecalcitol induced differentiation and reduced the basal OCR/ECAR ratio, a metric measuring the balance between mitochondrial respiration and glycolysis in cells (Figure 7D). This is consistent with decreased energetic demand from slower cell growth. Basal respiration, ATP production, maximal respiration, and spare respiratory capacity are all increased in inecalcitol treated cells. Basal respiration, the OCR prior to any inhibitor injection minus non-mitochondrial respiration, is increased with inecalcitol (Figure 7E). Maximal respiration, determined as the maximum OCR reached after FCCP injection, minus non-mitochondrial respiration, reflects the cell’s maximal electron transport chain capacity, and is increased with inecalcitol (Figure 7F). ATP production (ATP-linked respiration), calculated as the decrease in OCR after oligomycin injection, representing the portion of basal respiration coupled to ATP synthase activity, is increased in inecalcitol (Figure 7G). And finally, spare respiratory capacity, calculated as the difference between maximal respiration and basal respiration, indicates the ability of cells to respond to increased energetic demand, is also increased with inecalcitol (Figure 7H). All these metrics are reduced when autophagy was inhibited with BafA1 treatment. Similar results were obtained from MV4–11 cells (Figure S7). These data show that inecalcitol-induced myeloid differentiation improves mitochondrial function and metabolic flexibility in an autophagy-dependent manner.

## Discussion

We show that developmental stage of leukemia correlates with autophagic flux, where phenotypically primitive AML cells have lower autophagic flux while more differentiated monocytic cells have higher autophagic flux. Terminally differentiated myeloid cells, including neutrophils and macrophages, function in parasitic defense, which include the use of LC3-associated phagocytosis (LAP), ROS production, and degranulation (14, 61–63). These processes are mechanistically linked to autophagy and share many core components (12, 13, 15, 36, 64–66). Furthermore, autophagy is invoked during immune cell differentiation (13–16). How this relationship between autophagy and differentiation functions or persists in the context of AML is not clear. There are examples of differentiation induction invoking autophagy in acute promyelocytic leukemias (APL). The differentiation therapy drug all-trans retinoic acid (ATRA) is used to treat APL, and autophagy has been shown to target the oncogenic fusion protein PML-RARα for degradation (67, 68). In chronic myeloid leukemia (CML), upon acquiring imatinib resistance, activation of the ERK-mTOR axis suppressed autophagy and the degradation of the X-linked inhibitor of apoptosis protein (XIAP) is switched to proteasomal destruction. Restoring autophagic flux via mTOR inhibition restored both XIAP degradation and imatinib sensitivity (69). Thus, for these contexts, autophagy contributes to therapeutic efficacy, contrasting with our results.

In the case of AML, autophagy status correlates to sensitivity to Ven/Aza therapy, where high autophagy cells are more resistant to therapy. We demonstrate that this relationship remains true not only in stable differentiation-based subpopulations, but also during natural transient fluctuations in autophagy. Intriguingly, expression profiles from sorted transient autophagy high versus low MOLM-13 closely resembled that of previously published data comparing monocytic to primitive AML LSC, where Ven-resistant monocytic LSC, and in our case autophagy high cells, exhibit increased innate immune and inflammatory signaling (3). We also see a reduction in MYC hallmark pathways. MYC is a central metabolic regulator that drives biogenesis and has a well-established role in regulating autophagy, generally directly increasing expression of autophagy related genes including LC3, BECN1, ATG3, ATG5, and ATG7, to support tumor metabolism and survival under stress (42, 70–72). However, here we show evidence that MYC hallmark expression is reduced in autophagy high cells. MYC can in fact also suppress autophagy (40, 41, 72). Low MYC together with high stress and inflammatory signals is suggestive of a strong energy stress, where the anabolic pathways driven by MYC are suppressed while the energy salvaging autophagy pathways remain engaged. In AML, autophagy induction has been linked to drug resistance; for example, the pro-survival protein Bcl-2 inhibits apoptosis as well as autophagy. Thus, while Bcl-2 inhibition with venetoclax can induce apoptosis, its inhibition leads to induction of autophagy that can protect tumor cells from organelle damage and nutrient deprivation (73). Similarly, the apical autophagy kinase ULK1 is elevated in leukemic blasts compared to normal hematopoietic cells, and inhibition of ULK1 suppresses treatment-induced autophagy, overcomes adaptive drug-resistance, and synergizes with chemotherapy and emerging antileukemia agents like venetoclax (74).

We found that inecalcitol, known to induce differentiation in AML cells, was a potent inducer of autophagy. Vitamin D and analogs like inecalcitol are known differentiating agents and have been tested as differentiation therapy and even as a preventative cancer risk reduction treatment (75–78). Limited VDR expression has also been associated with impaired myeloid progenitor differentiation, and VDR agonists in combination with hypomethylating agents promotes leukemia stem cell exhaustion and decreases tumor burden in AML mouse models (47). These studies have led to clinical trials, such as NCT02802267, testing inecalcitol in combination with decitabine in AML. Vitamin D supplementation is administered as supportive care and has been shown to reduce complications of intense chemotherapy but not improve overall survival or outcomes (79).

Here we show that even transient induction of differentiation with inecalcitol increases Ven/Aza resistance dependent on the associated induction of autophagy. Similarly, driving differentiation by ectopic expression of CEBPA also induces autophagy and Ven/Aza resistance. We show that this myeloid differentiation-associated autophagy improves mitochondrial functional reserve and metabolic flexibility. So, despite the antiproliferative and differentiation effects of inecalcitol on AML, our studies here show differentiation of AML, and the corresponding increase in autophagy and OXPHOS, has protective effects against apoptosis-inducing Ven/Aza therapy. These results may explain the lackluster clinical efficacy with inecalcitol or vitamin D combination therapies. Mitophagy, a form of autophagy that selectively removes damaged and dysfunctional mitochondria, is known to improve OXPHOS and protect against mitochondrial apoptosis (80–84) and gives further credibility to the view that differentiation therapy must be paired with killing. Our studies also raise the potential concern that vitamin D therapy or supplementation could reduce responses of patients with AML to apoptosis inducing therapies such as those based on Venetoclax. More speculatively, we can envision therapeutic strategies that toggle treatments that preferentially target more primitive (e.g. Ven/Aza) or more differentiated (e.g. necroptosis-inducing) AML subpopulations, and even strategically incorporating treatments that promote differentiation. Finally, targeting OXPHOS has promise for therapeutically targeting AML (85–87). Our work here provides further support for disrupting the mitochondrial fuel supply for OXPHOS by inhibiting autophagy as a strategy to target AML.

## Supplementary Material

Supplementary Files

This is a list of supplementary files associated with this preprint. Click to download.

- MergedMenzeletalSupplementalFiguresMethods.pdf

## Figures and Tables

**Figure 1.**
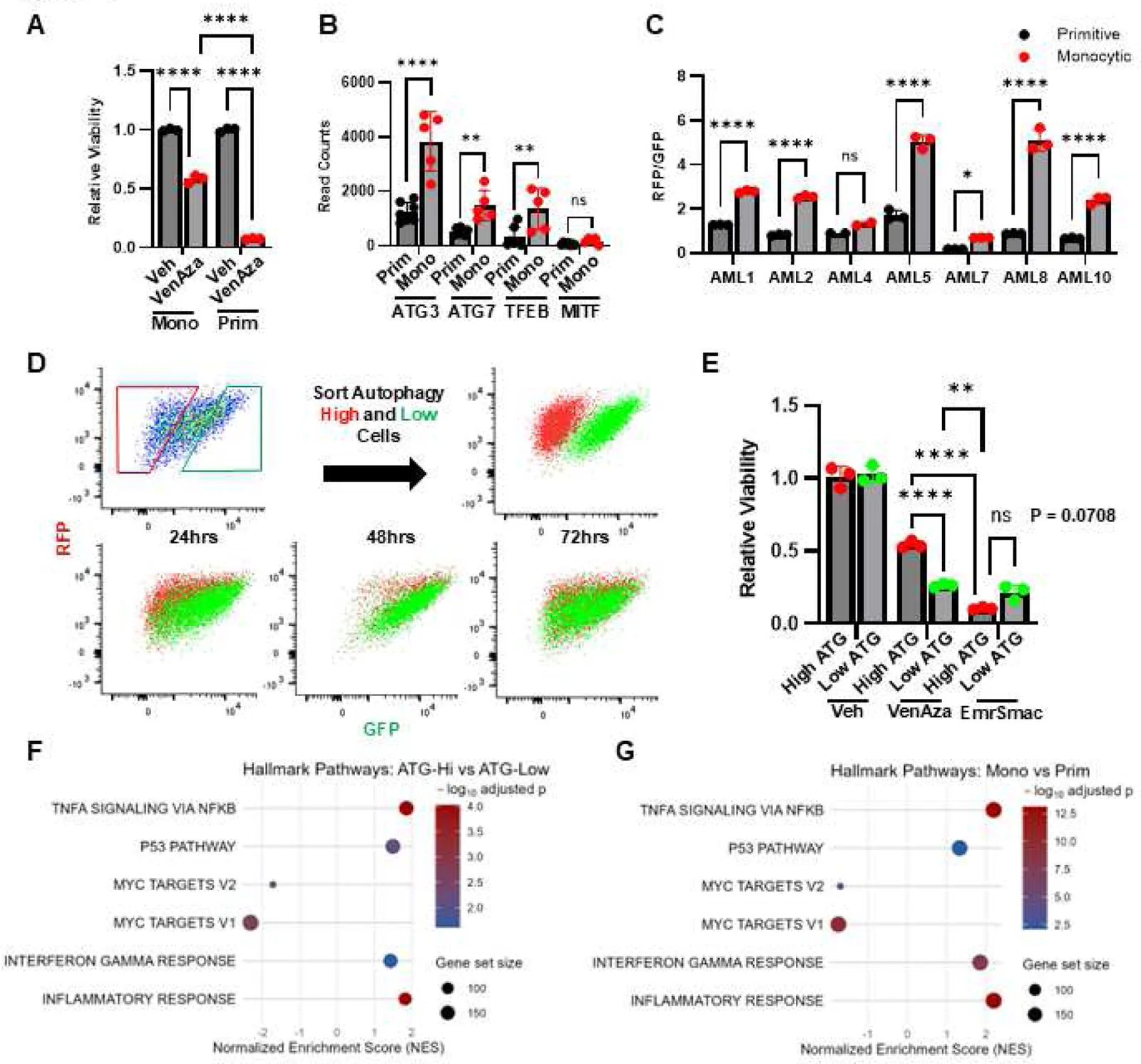
Both stable differentiation-based high autophagy and transient high autophagy are associated with Venetoclax resistance and inflammatory signaling. (A) Relative survival of primary AML1 subpopulations treated with 500nM Ven and 1.5μM Aza for 72hrs in vitro and analyzed by flow cytometry with differentiation markers to distinguish CD11b+, CD14+ monocytic cells, and CD11b-, CD14− primitive cells. (B) Read counts of select autophagy related transcripts in sorted VenAza resistant monocytic and Ven/Aza sensitive primitive cells from Pei et al. 2020 RNA-Seq data set. (C) Primary AMLs harboring the GFP-LC3-RFP autophagy reporter show that apoptotic resistant monocytic subpopulations exhibit higher autophagy (higher RFP/GFP). (D) MOLM13 cells harboring the GFP-LC3-RFP autophagy reporter sorted by high or low autophagy, approximately the top and bottom 15% RFP/GFP ratio respectively, show autophagic flux is transient and autophagy high and low cells converge within 48hrs. (E) Sorted high and low autophagy MOLM13 cells were treated with apoptosis inducing 100nM Ven and 1.5μM Aza or necroptotic inducing 5uM Emricasan with 50nM AZD5582 for 48hrs. (F) Select Hallmark Pathways from GSEA of sorted MOLM13 autophagy high (ATG-Hi) vs autophagy low (ATG-Low) RNA-Seq. (G) Select Hallmark Pathways from GSEA of monocytic vs primitive AML LSC RNA-Seq data from Pei et al. 2020. Error bars represent SEM, two-way ANOVA with Tukey’s multiple comparisons test; *, P < 0.05; **, P < 0.01; ***, P < 0.001; and ****, P < 0.0001.

**Figure 2.**
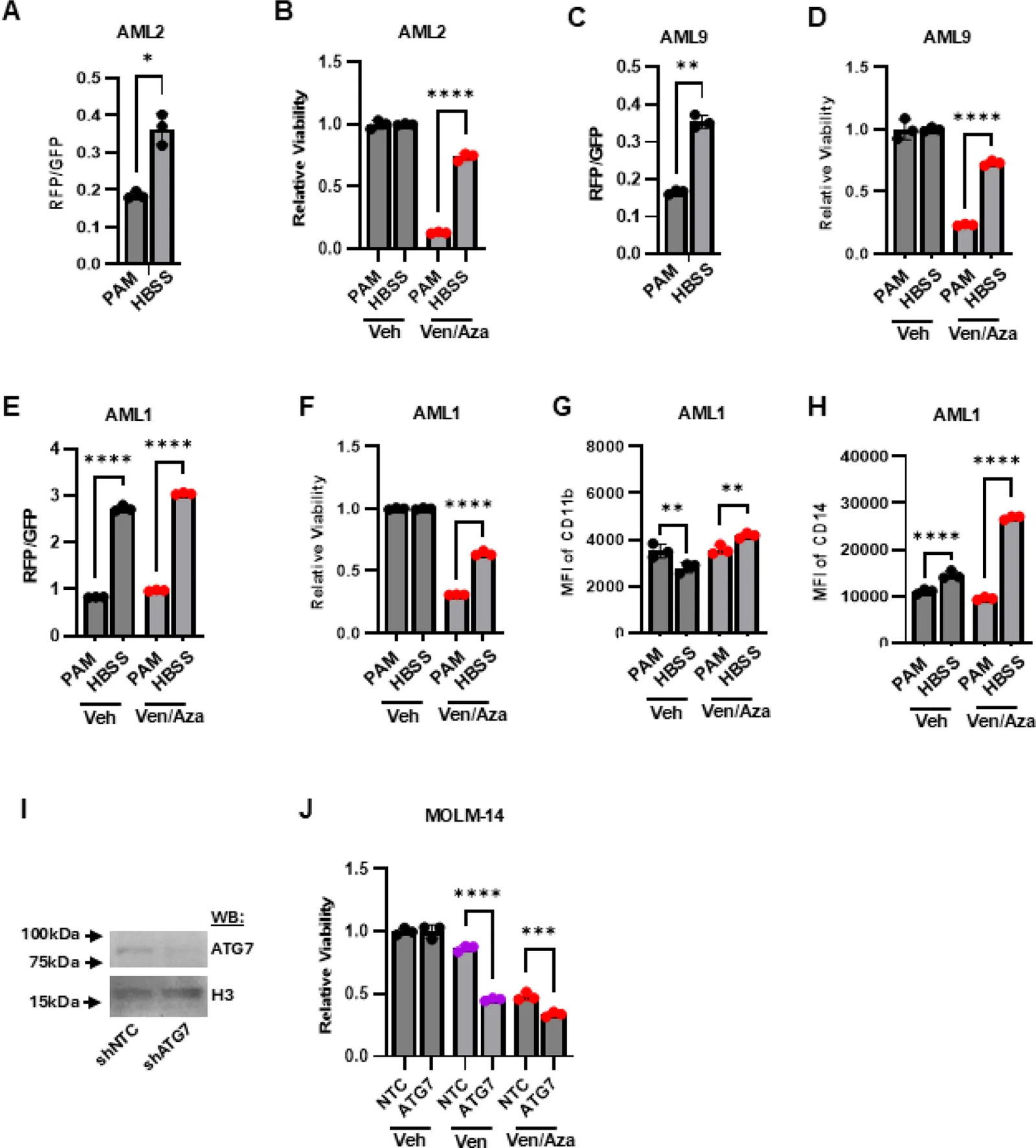
Starvation induced autophagy promotes VenAza resistance. (A, C, and E) Autophagic flux as measured from the RFP/GFP ratio of primary AML2, AML9, and AML1 resuspended in HBSS supplemented with 10% FBS and 2mM glutamine for 24hrs to simulate nutrient deprivation. (B, D, and F) Relative viability of AML either pretreated in HBSS media for 24hrs to simulate nutrient depravation (starve) or not, then resuspended into PAM and treated with 500nM Venetoclax and 1.5uM Azacitidine for 48hrs. (G) MFI of CD11b myeloid surface markers of cells in E and F. (H) MFI of CD14 myeloid surface markers of cells in E and F. (I) ATG7 Western blot of MOLM-14 cells transduced with lentiviral vector harboring shRNA against core autophagy gene ATG7 (pLKO.1 shRNA-ATG7-GFP) or nontargeting control pLKO.1 shRNA-NTC-GFP; Western blot of histone H3 as loading control. (J) Relative viability of MOLM14 cells harboring pLKO.1 shRNA-NTC-GFP or pLKO.1 shRNA-ATG7-GFP treated with 150nM venetoclax or 150nM venetoclax and 1.5uM azacitidine for 72hrs. Error bars represent SEM, two-way ANOVA with Tukey’s multiple comparisons test; *, P < 0.05; **, P < 0.01; ***, P < 0.001; and ****, P < 0.0001.

**Figure 3.**
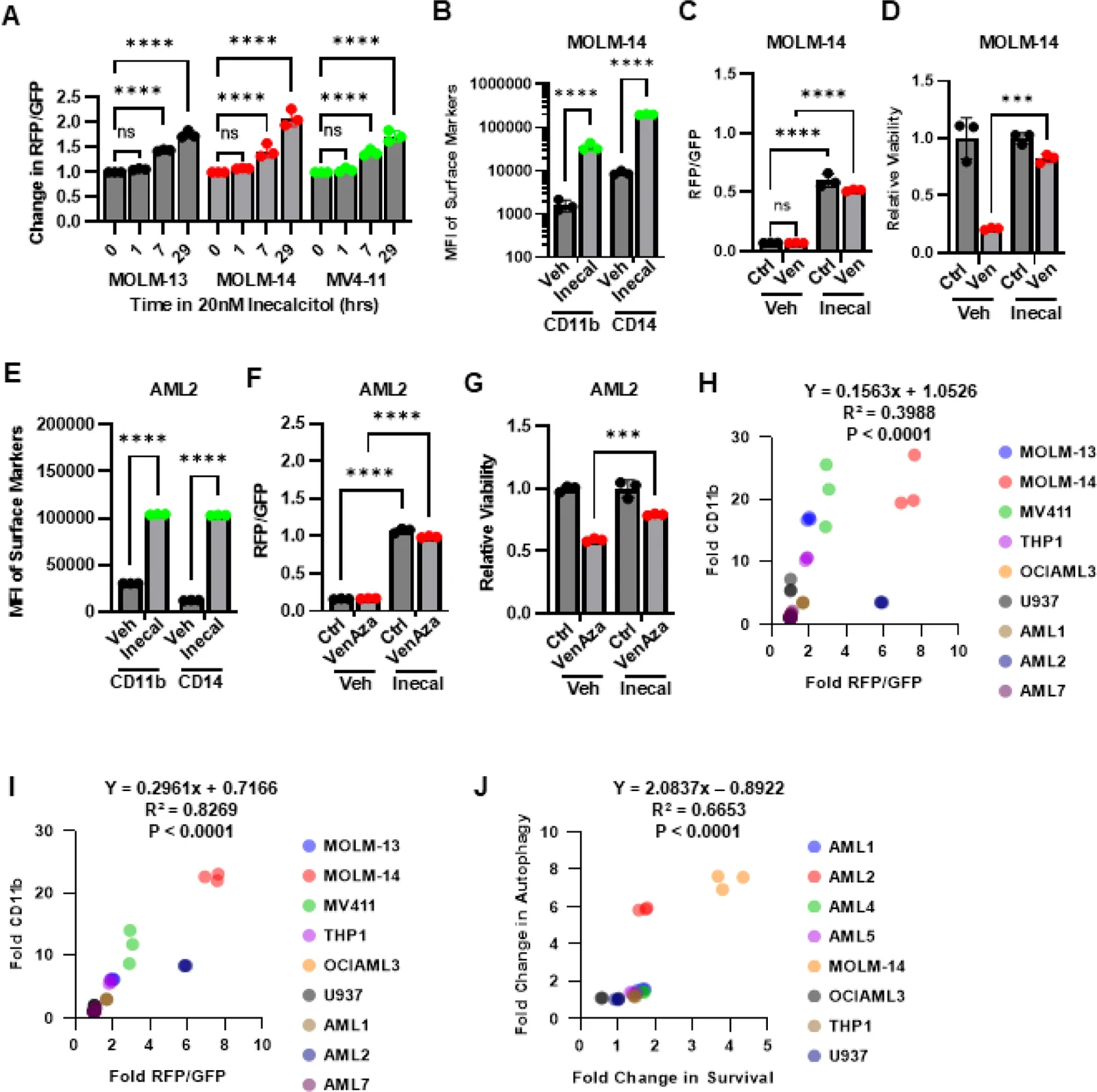
Vitamin D receptor agonist inecalcitol induces AML differentiation, increases autophagic flux and venetoclax resistance. **(A)** Autophagic flux time course as measured by fold change in MFI of the RFP/GFP ratio of AML cell lines MOLM-13, MOLM-14, and MV4–11 harboring the GFP-LC3-RFP autophagy reporter, treated with 20nM Inecalcitol for 1, 7, or 29 hrs measured with flow cytometry, showing increase in autophagic flux (higher RFP/GFP). **(B and E)** MFI of myeloid surface markers CD11b and CD14 of MOLM-14 and AML2 cells treated with 20nM Inecalcitol for 72 hrs. **(C and D)** MFI of RFP/GFP ratio of Molm14, AML2 cells treated with 20nM Inecalcitol for 72 hrs then treated with 500nM venetoclax (MOLM-14) or 500nM venetoclax and 1.5uM azacitidine (AML2) for an additional 48 hrs. **(D and G)** Relative viability of cells in C and F. **(H and I)** Fold change in autophagy by fold change in CD11b (I) and CD14 (J) of MOLM-13, MOLM-14, MV4–11, THP1, OCIAML3, U937, AML1, AML2, AML7. **(J)** Fold change in autophagy by fold change in viability of MOLM-14, THP1, OCIAML3, U937, AML1, AML2, AML4, AML5. Error bars represent SEM, two-way ANOVA with Tukey’s multiple comparisons test; *, P < 0.05; **, P < 0.01; ***, P < 0.001; and ****, P < 0.0001.

**Figure 4.**
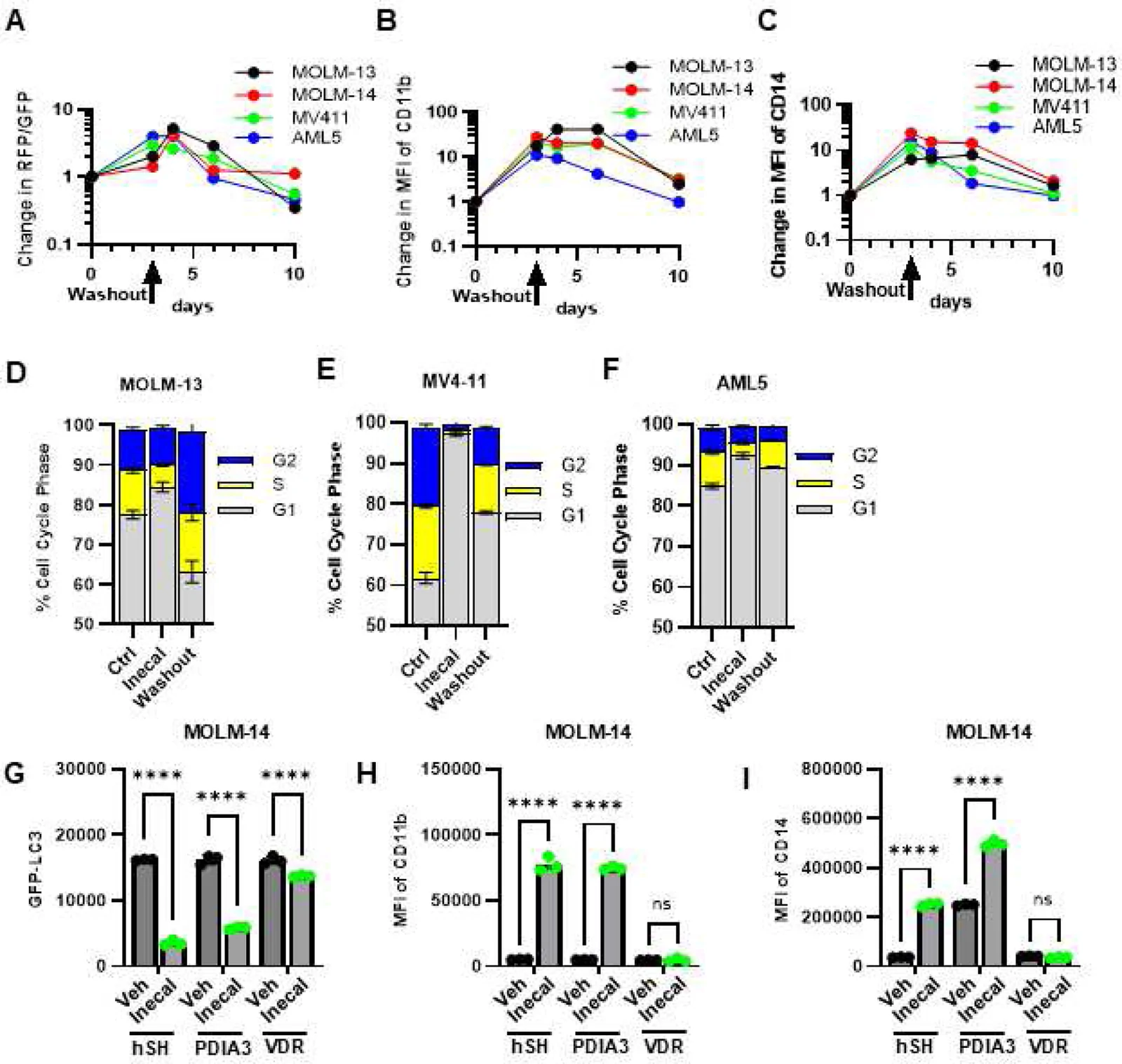
Effects of inecalcitol on autophagic flux, cell cycle, and AML differentiation are reversible and depend on VDR. (A) Fold change in MFI of RFP/GFP ratio of cell lines MOLM-13, MOLM-14, MV4–11, and primary AML0805 harboring the GFP-LC3-RFP autophagy reporter, treated with 20nM Inecalcitol for 3 days then washed out and allowed to recover for an additional 7 days. (B and C) Fold change of MFI of myeloid surface markers CD11b and CD14 of cells in A. (D, E and F) Cell cycle profiles of MOLM-13, MV4–11, and primary AML5 as measured by Hoechst 33342 staining, treated with 20nM Inecalcitol for 5 days then washed and allowed to recover for an additional 5 days. (G) MFI of GFP-LC3 of MOLM-14 cells harboring Cas9-Blast, the GFP-LC3 autophagy reporter, and LentiGuild-dTOM vectors for CRISPR mediated knockout of either VDR, PDIA3, or hSH (human safe harbor locus) treated with 20nM Inecalcitol for 72hrs. (H, and I) MFI of myeloid surface markers CD11b and CD14 of cells in G. Error bars represent SEM, two-way ANOVA with Tukey’s multiple comparisons test; *, P < 0.05; **, P < 0.01; ***, P < 0.001; and ****, P < 0.0001.

**Figure 5.**
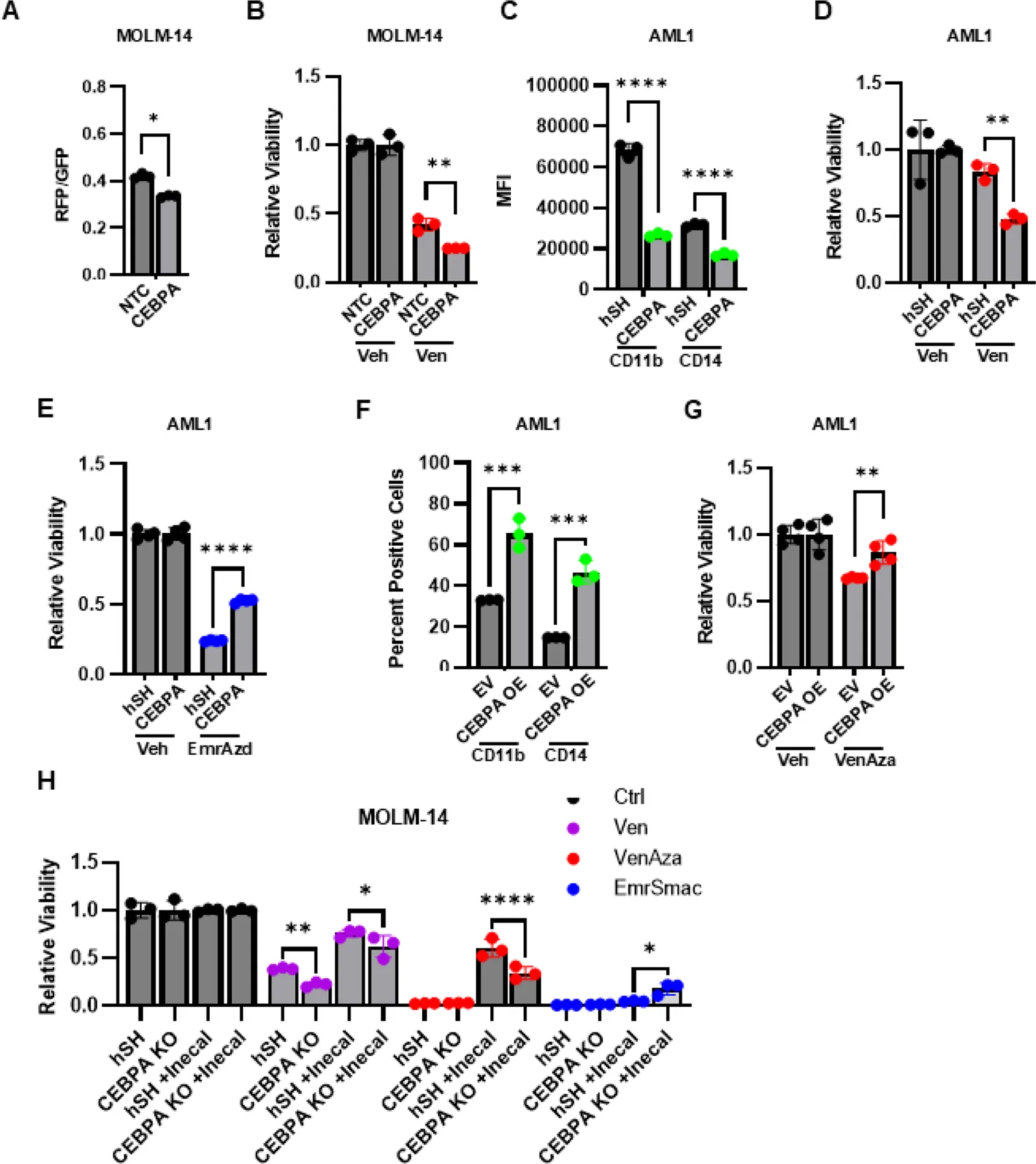
Driver of hematopoietic differentiation CEBPA controls autophagy and venetoclax resistance. (A) RFP/GFP ratio of MOLM-14 cells harboring the GFP-LC3-RFP autophagy reporter and Cas9-Blast, 5 days post CRISPR mediated deletion of CEBPA (KO) or non-targeting control (NTC) via lentiGuild-puro virus transduction. (B) Relative viability of cells in A treated with 500nM Venetoclax for 24hrs. (C) MFI of monocytic surface markers CD11b and CD14 in AML1 14 days post transduction with either the human safe harbor (hSH) lentiGuild-mCherry-hSH or lentiGuild-mCherry-CEBPA guide RNA expressing constructs, 2 days post-electroporation with 1.5 μg of Cas9 mRNA per 200 000 cells. (D) Relative viability of cells in C treated with 100nM Venetoclax for 48hrs. (E) Relative viability of cells in C treated with necroptotic inducing 5uM Emricasan with 50nM AZD5582 for 48hrs. (F) MFI of monocytic surface markers CD11b and CD14 in AML1 with ectopic expression of CEBPA (OE),14 days post transduction of pUltra-CEBPA-GFP. (G) Relative viability of cells in F treated with 500nM Venetoclax and 1.5uM Azacitidine for 48hrs. (H) Relative viability of MOLM-14 cells treated with or without 20nM Inecalcitol for 4 days prior to 48hrs treatment with 100nM Venetoclax, or 100nM Venetoclax and 1.5uM Azacitidine or 5uM Emricasan and 50nM AZD5582. MOLM-14 cells harboring Cas9-Blast were transduced for lentiviral CRISPR-mediated deletion of CEBPA (KO) or human safe harbor (hSH) via lentiGuild-mCherry 4 days prior to drug treatment. Error bars represent SEM, two-way ANOVA with Tukey’s multiple comparisons test; *, P < 0.05; **, P < 0.01; ***, P < 0.001; and ****, P < 0.0001

**Figure 6.**
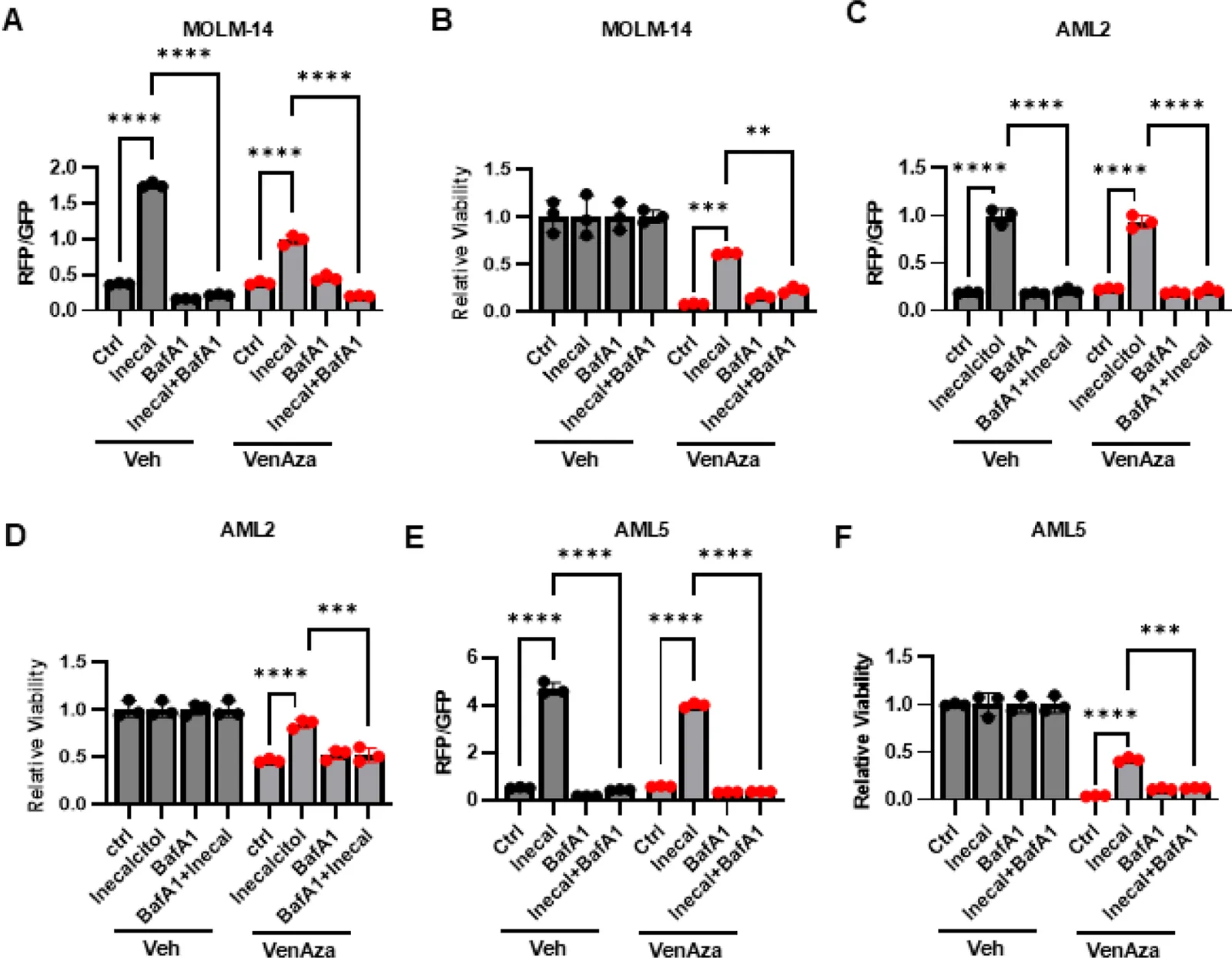
Inhibition of autophagy negates protective effects of inecalcitol induced myeloid differentiation. (A, C, E) RFP/GFP ratio of autophagic flux reporter in MOLM-14, AML2, and AML5 pre-treated with vehicle (Ctrl) or 20nM Inecalcitol for 72hrs and then treated for an additional 48hrs with 500nM Ven and 1.5μM Aza (Ven/Aza), with or without 100nM Bafilomycin A1 (BafA1) to block autophagy. (B, D, F) Relative viability of cells in A, C, and E. Error bars represent SEM, two-way ANOVA with Tukey’s multiple comparisons test; *, P < 0.05; **, P < 0.01; ***, P < 0.001; and ****, P < 0.0001.

**Figure 7.**
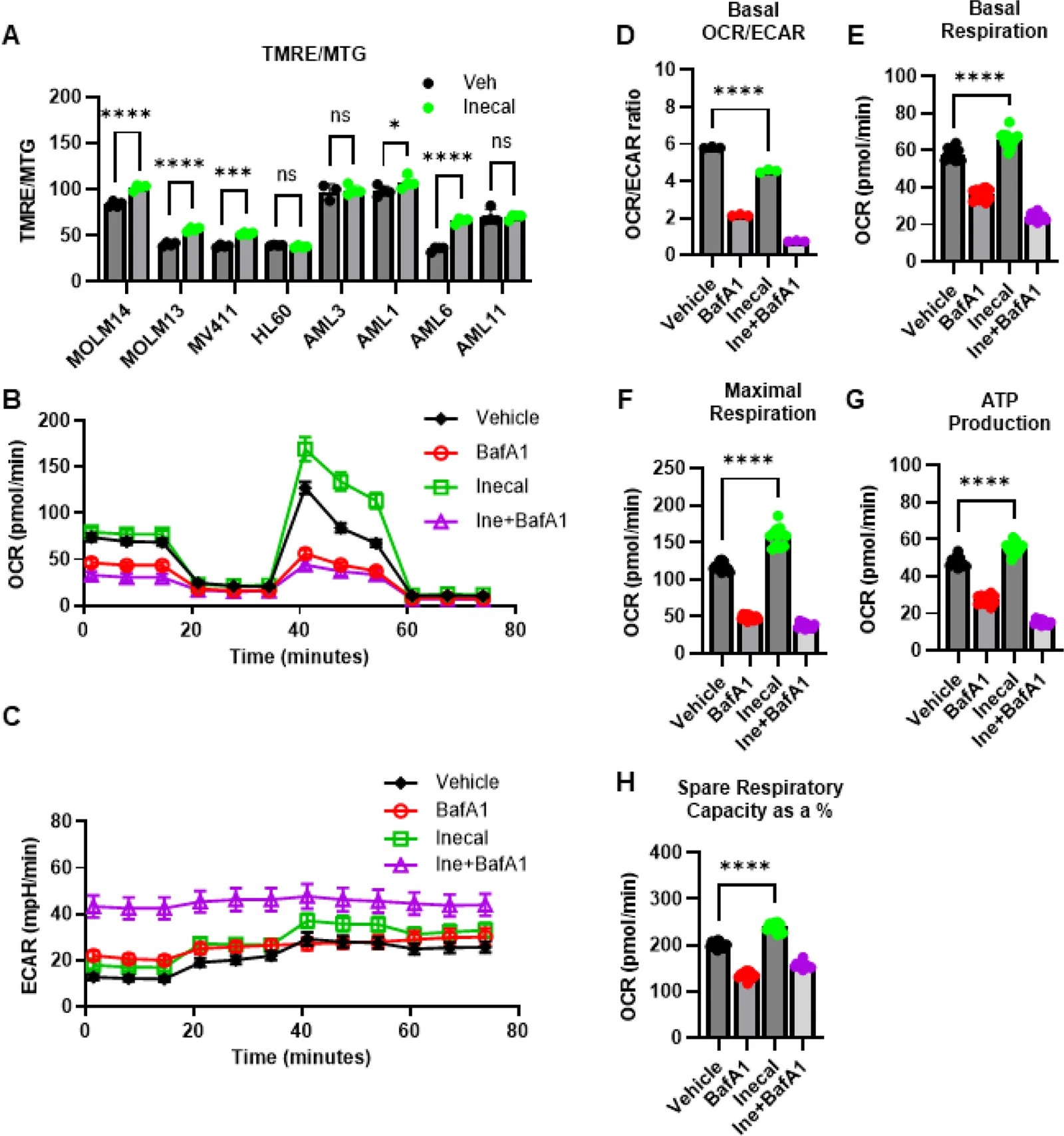
Differentiation-associated autophagy improves mitochondrial reserve capacity and metabolic flexibility. (A) Mitochondrial transmembrane potential normalized to mitochondrial mass as measured by flow cytometry after 30 minutes of TMRE and MitoTracker Green staining of primary AML and cell lines treated with vehicle or 20nM Inecalcitol for 4 days. (B) Agilent Seahorse XF Cell Mito Stress Test presenting Oxygen Consumption Rate (OCR) and (C) Extracellular Acidification Rate (ECAR) of AML1 treated with 20nM Inecalcitol for 4 days or vehicle, with or without 100nM Bafilomycin A1 for the last 18hrs just prior to replating cells onto CellTak coated Agilent Seahorse XF Assay plate. Oligomycin, FCCP and Rotenone/AntimycinA treatment were at 20, 40 and 60 minutes respectively. (D) OCR/ECAR at basal time points, pre-oligomycin treatment, of AML1. (E) Basal respiration of cell in B. (F) Maximal respiration of cell in B. (G) ATP production of cell in B. (H) Spare respiratory capacity as a percent of cell in B. Error bars represent SEM, two-way ANOVA with Tukey’s multiple comparisons test; *, P < 0.05; **, P < 0.01; ***, P < 0.001; and ****, P < 0.0001.

## Data Availability

RNA-seq data will be deposited in GEO and made available prior to publication. Any other raw data or materials (such as constructs) will be made freely available to other researchers upon request.
